# Biomarkers of Frailty: miRNAs as Common Signatures of Impairment in Cognitive and Physical Domains

**DOI:** 10.3390/biology11081151

**Published:** 2022-07-31

**Authors:** Serena Dato, Paolina Crocco, Francesca Iannone, Giuseppe Passarino, Giuseppina Rose

**Affiliations:** Department of Biology, Ecology and Earth Sciences, University of Calabria, 87036 Rende, Italy; paolina.crocco@unical.it (P.C.); francesca.iannone@unical.it (F.I.); giuseppe.passarino@unical.it (G.P.); pina.rose@unical.it (G.R.)

**Keywords:** miRNA, frailty, physical domain, cognitive domain, biomarkers, multisystemic impairment

## Abstract

**Simple Summary:**

A growing number of studies have focused their attention on the potential role of microRNAs (miRNA) as biomarkers for several diseases. However, very few evaluated the role of miRNAs in the aetiogenesis of frailty, a multidimensional geriatric syndrome, characterized by an individual and dynamic state of impairments in one or more domains, such as physical, cognitive, psychological, and social. In this review, we first provided an overview on the different frailty domains, current assessment tools and plasma/blood biomarkers. Then, we collected the evidence linking changes of miRNAs expression to impairment of frailty in physical and cognitive domains, with the ultimate aim of finding those that are common. In silico analyses prioritized ten top-ranked miRNAs and their targets, the three most significant regulating processes involved in inflammation and energy homeostasis pathway. Such miRNAs, through the integration with existing markers, may be useful for an early and accurate diagnosis of frailty in the elderly population.

**Abstract:**

The past years have seen an increasing concern about frailty, owing to the growing number of elderly people and the major impact of this syndrome on health and social care. The identification of frail people passes through the use of different tests and biomarkers, whose concerted analysis helps to stratify the populations of patients according to their risk profile. However, their efficiency in prognosis and their capability to reflect the multisystemic impairment of frailty is discussed. Recent works propose the use of miRNAs as biological hallmarks of physiological impairment in different organismal districts. Changes in miRNAs expression have been described in biological processes associated with phenotypic outcomes of frailty, opening intriguing possibilities for their use as biomarkers of fragility. Here, with the aim of finding reliable biomarkers of frailty, while considering its complex nature, we revised the current literature on the field, for uncovering miRNAs shared across physical and cognitive frailty domains. By applying in silico analyses, we retrieved the top-ranked shared miRNAs and their targets, finally prioritizing the most significant ones. From this analysis, ten miRNAs emerged which converge into two main biological processes: inflammation and energy homeostasis. Such markers, if validated, may offer promising capabilities for early diagnosis of frailty in the elderly population.

## 1. Introduction

### 1.1. The Frailty Domains

Frailty is a common clinical syndrome in older adults characterized by a multisystem impairment which ranges from musculoskeletal, to pulmonary, cardiovascular and neurological systems and by a marked vulnerability to adverse health outcomes, including an increased risk of disability, admission to long-term care and increased mortality [1,2]. Aging, which causes a progressive decrease in the physiological reserves and an overall loss of homeostasis, is the main risk factor for frailty. The pace of this progressive decay is accelerated by genetic factors, epigenetic events and environmental stressors [3].

**Figure 1:**
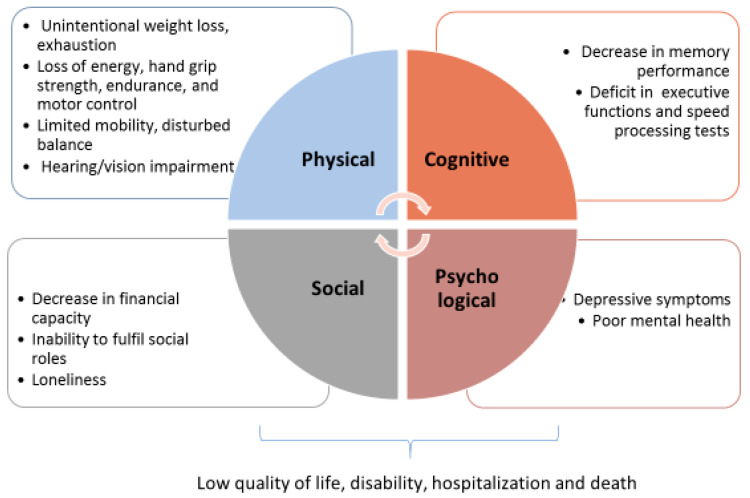
The multi-domain model of frailty.

Literature still lacks a consensus for a comprehensive definition of frailty, although the classification of frailty which most of all embraces its complex nature is that of a multi-domain phenotype, firstly proposed by Abellan Van Kan et al. [4] and further developed by many authors [5,6,7,8,9]. As outlined in Figure 1, the multi-domain model includes physical, cognitive, psychological and social domains of frailty, while Box 1 describes the criteria generically used for the classification of physical, cognitive and psychological/social declines, applied by many frailty tools such as that of Fried et al. [10], Rockwood and Mitnitski [11], Gobbens et al. [12], Peters et al. [13], Morley et al. [14] and Wieland and Hirth [15]. The tools comprehend tests which can be used as endophenotypes (defined as quantitative biological traits which reflect the function of a discrete biological system), closely related to the specifical domain respect to the broad phenotype [16,17,18].

Box 1 **Criteria used for the classification of impairments in the physical, cognitive and psychological/social domains of frailty, applied by many frailty tools as described in the text.**

**The physical domain**
Physical frailty was reported by Maxwell and Wang [8] as “characterized by gradual loss of energy, strength, endurance, and motor control”. It is defined basically considering ≥4 of 8 criteria, reflecting the screening of muscle health and functional status: unintentional weight loss, exhaustion, strength, perceived health, walking, balance, hearing and vision impairments [19]. A series of socio-demographic, lifestyle, and health-related factors have been shown to be associated with physical frailty, such as age, female sex, cardiovascular diseases, multimorbidity, BMI, and smoking.
**The cognitive domain**
Cognitive impairment in absence of dementia is considered a relevant domain of frailty. Cognitive impairment is defined as <10th percentile on global cognitive functioning, detected with cognitive tests, such as the MMSE (Mini Mental State Examination) score [20] and the cognitive abilities screening instrument (CASI) [21].
**The psychological and social domains**
Psychological frailty is defined on the base of two criteria such as depressive symptoms and mental health, and is measured by Geriatric Depression Scale and Mental Health Inventory 5 (MHI-5) [22]. A higher psychological frailty risk is associated with the female sex, low educational level, smoking, a short sleep duration and multi-morbidity, while being married, a long sleep duration and being physically active are normally associated with a lower risk of being psychologically frail.
Social frailty is measured as ≥2 of 3 criteria among loneliness, social support and social participation.

Common to all the frailty assessment tools is the physical domain, while the cognitive domain is included in only 50% of them. This because in the past there was still some uncertainty about the relationship between frailty, cognitive impairment and dementia, so that some authors exclude people severely cognitively compromised in the analyzed cohorts. Recently, cognitive impairment was recognized as a significant determinant of frailty and consequently a novel target for the prevention [23].

Growing evidences suggest that the different frailty domains are not independent entities, but share subcellular pathophysiological mechanisms, not only increasing the vulnerability and frailty prevalence [24] but also forming a substrate for the development of chronic age-related diseases, such as Alzheimer diseases (AD), where physical frailty was proposed as a non-cognitive manifestation that precedes the onset of dementia [25]. In addition, social and physical frailty were considered as a risk factor for the development of the other, considering that a decline in social roles precedes the activity of daily living disabilities among community-dwelling independent older adults, with an impact on the mortality risk [26,27].

Although at a population level the overlap among impairments in different frailty domains can be limited to a small proportion of the oldest population, with around 17% of people found frail at two or more domains [19], evidences were reported that multi-domain interventions are able to improve health status in elderly people at-risk from the general population [28]. The co-occurrence of physical, cognitive and emotional decline was named triad of impairment (TOI) and it was used in the past as a surrogate marker of frailty in some studies, such as the Cardiovascular Health study [10] and Aberdeen Birth Cohort study [29].

**Figure 2:**
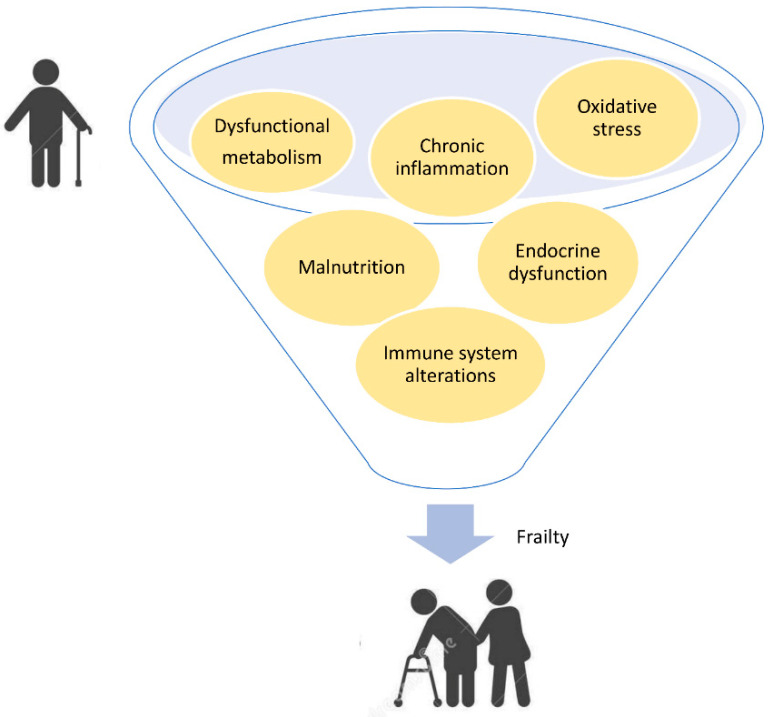
Impaired biological mechanisms shared among different frailty domains.

The overlap among different phenotypic domains of frailty account for pathogenic pathways shared among multiple districts, which provide a background prone to dysfunction, driving the accumulation of deficits which concur with frailty development. By filtering the extensive literature [30,31,32] (and references therein), seven most-cited and replicated biological mechanisms dysregulated in frailty can be retrieved, which are common to different domains, as schematized in Figure 2.

Such deficiencies, such as chronic inflammation, immune and endocrine impairment, dysregulated metabolism, oxidative stress and malnutrition, are common and interconnected denominators observed in both physical and cognitive decline status, occurring in a very early step of frailty onset and contributing to its progression.

### 1.2. Current Biomarkers of Frailty

**Figure 3:**
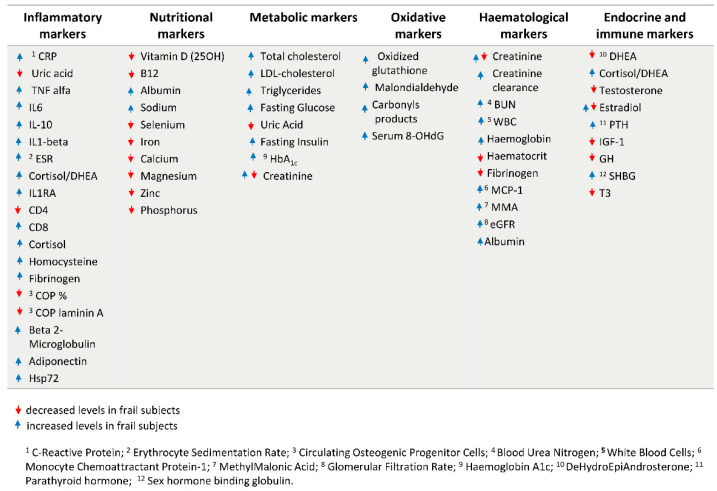
Circulating biomarkers of frailty divided in six sets of physiological parameters. The red and blue arrows indicate if increased or decreased levels were found in frail subjects, according to literature evidence, as reported in the text.

The identification of the above biological hallmarks has driven the research on the identification of circulating biomarkers whose screening, in association with a complete geriatric assessment, including cognitive and physical tests, can be helpful for a primary frailty diagnosis of the patient. Figure 3 [33,34,35,36,37] resumes the most common identified blood and biochemical markers of frailty covering different sets of physiological parameters, which includes inflammatory markers [38] oxidative markers [39,40], nutritional and metabolic markers, hematological markers, endocrine and immune markers [36].

However, while for some of them the association with frailty is clear, in other cases their efficiency as prognostic markers of frailty is still under discussion [41,42]. This is the case for creatinine, whose levels can reflect both kidney insufficiency and sarcopenia: thus, by using creatinine as a biomarker of frailty alone, a misidentification of frail subjects may occur [43].

Some biomarkers, such as endocrine ones, are age- and sex-specific, with different and sometimes opposite trends of association observed in males and females. For example, some studies reported that in men aged 65–90 years higher estradiol levels were associated with a decreased frailty [44], while in females higher estradiol levels were associated with increased frailty up to the age of 79, but not thereafter (Carcaillon et al. [45] for the Spanish Toledo Study for Healthy Aging). Moreover, an interaction between hormones and inflammation was found in women with CRP (C-Reactive Protein) levels. Thus, even though it is difficult to compare different studies, due to the application of different frailty measures, these evidences suggest that the intertwining among different physiological systems can drive the associations with frailty phenotype, further confirming the importance of considering the whole set of markers in an integrated approach [46,47].

## 2. Search for Novel Biomarkers of Frailty: The Role of miRNAs

Novel biomarkers of frailty can be reasonably chosen among factors acting in pathophysiological mechanism common to the physical and cognitive domains, and should be highly sensitive to minimum changes in physiological conditions. Such markers should be integrated with those previously discussed, to complete the picture of changes in the physiological status consequent to the occurrence of frailty. In this context, the evaluation of circulating microRNAs (miRNAs) was proposed as non-invasive diagnostic biomarkers of frailty, potentially regulating very large sets of genes (even hundreds of putative gene targets are known) and targeting different pathways at the same time [48].

MiRNAs are small non-coding single-stranded RNAs approximately 21–25 nucleotides long, regulating gene expression by binding to complementary messenger RNAs (mRNAs) and preventing the production of the corresponding protein [49]. MiRNA biogenesis is a step-wise process which starts with pri-miRNAs, long primary transcripts produced by RNA polymerase II and then processed into a pre-miRNA (~70- to 120-nucleotide-long) by a multiprotein complex containing the nuclear RNase III enzyme, known as Drosha. Then, this pre-miRNA is exported into the cytoplasm, to be processed into a mature duplex (~18- to 23-nucleotide-long) by the RNase III Dicer-1. A ribonuclear-protein complex, called RNA-induced silencing complex (RISC), composed by the guide strand of the duplex along with Argonaute proteins, directs to target mRNA and through sequence complementarity causes its translational repression [50]. A “seed region” (nucleotides 2 to 8) at the 5′ end of the mature miRNA can mediate the recognition of the mRNAs target site [51], usually by binding the target sequences at the 3′ UTR but sometimes also in 5′ UTR and open reading frame [52]. Each miRNA targets hundreds of transcripts, regulating fundamental cell processes such as proliferation, apoptosis, differentiation, migration, metabolism and stress response [53]. In addition to intracellular miRNAs, circulating extracellular miRNAs have been detected in different biofluids including blood, plasma, serum, saliva, urine and pleural effusions [54]. They can circulate as free molecules or be bound to carriers such as low-density lipoproteins (LDL), high-density lipoproteins (HDL), ribonucleoproteins and extracellular vesicles (EVs) [54]. In relation to different pathophysiological conditions, biofluids can have specific circulating miRNAs [55].

Several authors analyzed miRNA levels in pathological conditions [56,57,58] and in relation to aging [59,60,61,62,63], further supporting the use of miRNA panels as potential diagnostic biomarkers. A critical role of these small molecules has been documented relative to the frailty-associated phenotypes, i.e., in muscle development and homeostasis [64], but also in neuronal processes such as brain development, synaptic plasticity, and learning and memory functions [65]. A study of network biology, by analyzing the interactome of frailty-related genes, prioritized 10 miRNA markers indirectly associated with frailty through the association of their targets [66]. To date, only three studies directly evaluated changes in blood plasma miRNAs in frail/non frail subjects [67,68,69]. Rusanova measured levels of the inflammation-related miRNAs, miR-21, miR-146a, miR-223, and those of miR-483, associated with the control of melatonin synthesis, reporting an association between the expression of miR-21 with the presence of frailty [67]. Ipson and co-workers, instead, examined the levels of plasma-derived exosome miRNA and identified eight miRNAs significantly enriched in frailty subjects: miR-10a-3p, miR-92a-3p, miR-185-3p, miR-194-5p, miR-326, miR-532-5p, miR-576-5p, and miR-760 [68]. Interestingly, many of these markers modulate on/off switching of crucial cellular mechanism involved in frailty, such as miR-194-5p, which is both associated with cellular senescence and ROS production [70] and was reported to regulate muscle cell homeostasis [71]. Very recently, Carini and co-workers [69] carried out a larger study by profiling a total of 41 frail/non frail subjects for a miRNA set of 2654 markers. They found two miRNAs downregulated in the frailty group, namely miR-101-3p and miR-142-5p, both previously associated with oxidative stress-induced apoptosis and immune-inflammatory responses. As a whole, these researches indicate miRNAs mechanistically involved in the aetiogenesis of frailty, pinpointing the main pathways (as inflammation or ROS production) in common among different frailty domains, thus making them good candidates as frailty biomarkers.

In the following sections we reviewed the knowledge on miRNAs associated with physical and cognitive decline. In particular, we considered the literature that found associations between miRNAs and phenotypes related to physical frailty, such as muscle loss, sarcopenia, cachexia, or participating in the proliferation and differentiation of myogenic progenitors and myotubes, applying keywords such as “sarcopenia”, “physical impairment at old age”, “physical frailty”, “muscle loss” and “skeletal muscle decline”. In the case of the cognitive domain, we used keywords such as “cognitive decline”, “cognitive impairment” and “neurodegeneration”. Furthermore, for the retrieval of the list of relevant miRNAs, we excluded studies carried out in model organisms, focusing on human studies (cells, tissues or individuals) but excluding those related to severe pathological age-related conditions. In the case of cognitive domain of frailty, because our interest was to identify early markers of neurodegeneration, we considered early stages of Mild Cognitive Impairment (MCI).

## 3. MiRNAs as Biomarkers of the Physical Domain of Frailty

By reviewing the literature on phenotypes related to physical frailty, we identified a total of 57 miRNAs associated with physical phenotypes, resumed in Table S1 [67,68,69,72,73,74,75,76,77,78,79,80,81,82,83,84,85,86,87,88,89,90,91,92,93,94,95,96,97,98,99,100,101,102,103,104,105,106,107,108,109,110,111]. An inspection of their characteristics revealed that they belong to three main groups: muscle-related miRNAs, inflamm-miRNAs and mitochondrial miRNAs (mitomiRNA).

Among muscle-related miRNAs a sub-group, classified as myomiRs and comprising miR-1, miR-206, miR-208a, miR-208b, miR-133a, miR-133b, miR-486 and miR-499 [112] has been extensively investigated. These miRNAs regulate myoblast proliferation, differentiation and regeneration i.e., increasing the expression of targets such as the myogenic factors MYOG (myogenin), MYF5 (myogenic factor 5), MYOD1 (myogenic differentiation 1) and PAX7 (paired box protein 7), in order to induce muscle regeneration and to prevent fibrosis [113]. Moreover, beyond acting at muscle level, some of them can exert additional functions such as cell fate regulation, chromatin remodeling and oxidative stress control [57]. They can regulate, or be regulated by, factors involved in the IGF-1/Akt/mTOR signaling pathway, known to control skeletal muscle protein synthesis and muscle protein breakdown, processes controlled by anabolic stimuli, such as physical activity and nutritional status [88,94,97,114,115].

Many miRNAs related to physical frailty are involved in inflammation, which is recognized as the underlying pathway in sarcopenia and muscle loss. The best known are miR-21 and miR-146a, proposed as inflamm-miRNA owing to their ability to master (NF-κB)-driven inflammatory pathways [116]. MiR-21 was proposed by Rusanova et al. [67] as a biomarker of human muscle frailty. Its levels were found to correlate with AOPP (Advanced oxidation protein products) levels, mediators of the proinflammatory effect of oxidative stress [117].

MiR-146a is one of the most prevalent miRNAs in the literature in instances of chronic inflammatory disorders and oxidative stress, both in the muscle [118,119] and in the brain [120] and is a regulator of osteogenesis and angiogenesis [121].

Other families of miRNAs related to both physical frailty and inflammatory status are miR-19 and miR-181 [122,123].

Finally, some miRNAs listed also regulate mitochondrial functions and are known as mitomiR [124]. Most of them are nuclear-encoded; some mitomiRs modulate mitochondrial function by binding mRNA in the cytoplasm (examples can be found among miR-27a, miR-34, mir-155 or miR-181a), while others are imported into mitochondria as part of RISC or pre-RISC and target mitochondrial-encoded mRNA (i.e., mir-151a and miR-181c).

## 4. MiRNAs as Biomarkers of the Cognitive Domain of Frailty

Table S2 [68,69,120,125,126,127,128,129,130,131,132,133,134,135,136,137,138,139,140,141,142,143,144,145,146,147,148,149,150,151,152,153,154] summarizes the 43 miRNAs reported in association with cognitive impairment/decline in older adults.

Actually, it is known that the brain expresses about 70% of experimentally detectable miRNAs [155].

The critical role of miRNAs in the central nervous system was demonstrated in model organisms, where the disruption of the miRNA biogenesis machinery [156,157,158] or the knock-out of specific miRNAs produced worsened long-term memory, enhanced Aβ burden, and increased tau pathology, revealing a possible influence of miRNAs on many genes of the tau subnetwork [159,160,161]. As for humans, in recent years two papers emerged with evidence for the discrimination power of miRNAs in respect to cognitive decline in old age. First, Kondo and collaborators (2019) found a positive correlation between low serum levels of miR-20a, miR-27a, and miR-103a and MMSE scores in Japanese individuals with early-stage cognitive decline [126]. After, Gullet et al. (2020) by an *in-silico* analysis proposed three miRNAs (miR-140-5p, miR-197-3p, miR-501-3p) as blood-based biomarkers of cognitive aging, and top-ranked predictors of multiple cognitive outcomes in healthy older adults [146].

Pathways targeted by multiple miRNAs include Aβ genesis, regulation of AMPAR subunits, autophagy homeostasis, apoptosis, microglial activation, NF-κB signaling, blood-brain barrier maintenance, synaptic plasticity and neurogenesis [120].

Interestingly, many miRNAs related to cognition also have roles in inflammation, confirming the inflammatory pathway as a major component of neurodegenerative diseases and a plausible mechanism at the crossroad between several frailty domains. Among them, some were addressed by some authors as NeurimmiRs (the best known are miR-9, miR-21, miR-124, miR-132, miR-135, miR-146a, miR-155, miR-186, miR-223, in addition to the miR-29 family) affecting both immune and neuroinflammatory processes [162]. The evidence documenting their role in different pathological conditions characterized by cognitive decline demonstrates the major role of miRNAs in the neuroimmune interface, acting as ‘negotiators’ between these two interacting compartments, through direct or indirect alterations of neuron-glia and/or brain-to-body signaling [162]. Considering the importance of the network nervous–endocrine–immune system in maintaining the overall homeostasis [163], these miRNAs may be at the crossroad between the cognitive domain and the other domains of frailty.

## 5. In Silico Analysis of Shared miRNAs between Cognitive and Physical Domains

**Figure 4:**
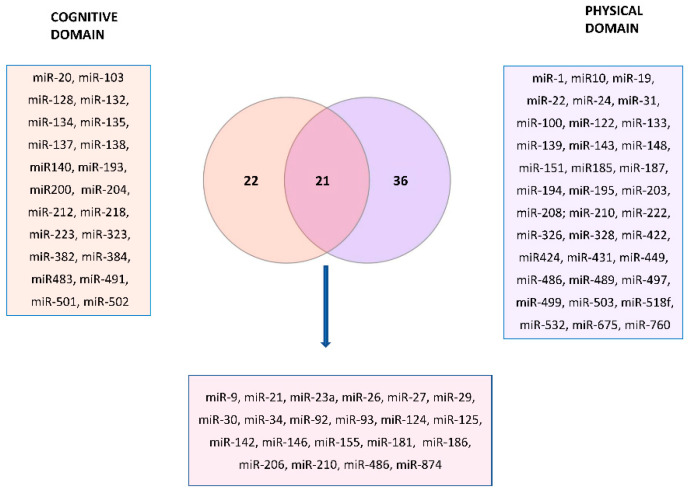
Venn diagram highlighting the (21) miRNAs markers shared between the cognitive and physical domains of frailty (listed in the violet square). The list of markers belonging to the cognitive domain (22) and physical domain (36) is also reported in the pink and indigo squares.

Figure 4 represents the Venn diagram describing the overlapping miRNAs between cognitive and physical domains, indicated in bold in the Tables S1 and S2.

There are 22 miRNAs related exclusively to the cognitive domain of frailty and 36 only to the physical domain, while 21 were common. We performed miRNA-target enrichment of the 21 miRNAs identified by a target prediction tool, MIENTURNET (http://userver.bio.uniroma1.it/apps/mienturnet/, accessed on 11 July 2022) [164] by using experimentally validated (miRTarBase) miRNA-target interactions for discover the targets of the candidate miRNA list. With an FDR cut-off <0.05, and a minimum number of two interactions of gene-miRNAs, we retrieved 55 genes significantly associated with those miRNA markers (Table 1).

**Figure 5:**
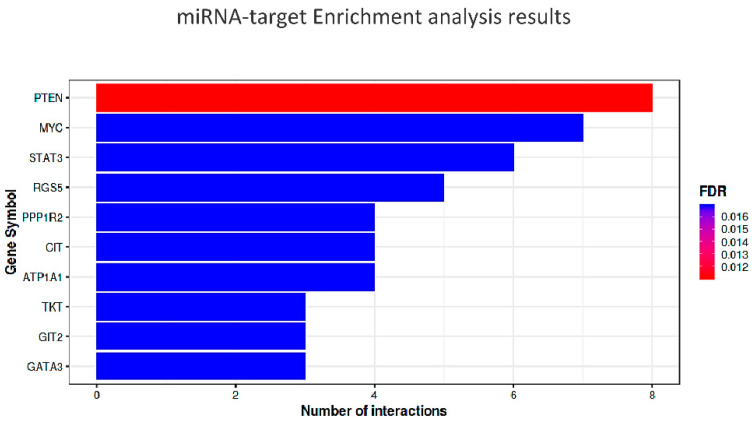
Bar plot of the results of the enrichment analysis, obtained by using miTarBASE as reference dataset. In the Y-axis are reported the top ten target genes of the submitted miRNAs, while the X-axis represents the number of miRNAs targeting them. The color code reflects the adjusted *p*-values (FDR) increasing from red to blue. The threshold for the minimum number of miRNA-target interactions was set up to 2.

The top ten most significant targets (minimum of three gene-miRNA interactions and *p* < 4.5 × 10^−5^) are indicated in italics and represented in the diagram of Figure 5.

**Figure 6:**
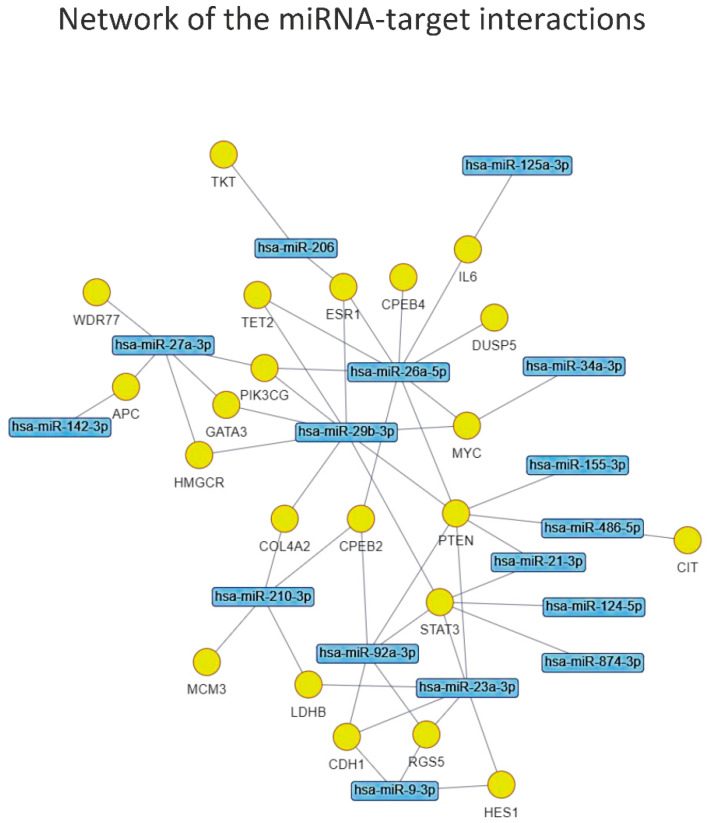
Graphical representation of the network of miRNA-target interactions identified by the enrichment analysis.

The network of miRNA-target interactions identified by the enrichment analysis (Figure 6) obtained by applying the same FDR < 0.05 allowed us to prioritize the most interesting miRNAs to submit for the functional enrichment analysis, which were: miR-26a-5p, miR-29b-3p, miR-23a-3p, miR-27a-3p, miR-92a-3p, miR-210-3p, miR-9-3p, miR-206,-miR-21-3p and miR-486-5p.

**Figure 7:**
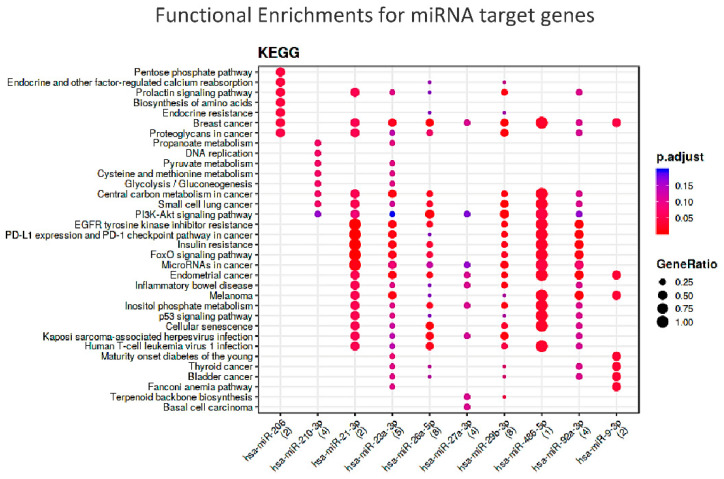
Functional Enrichments for miRNA target genes according to KEGG db. N.B.: The colors of the dots represent the adjusted *p*-values (FDR), whereas the size of the dots represents gene ratio (i.e., the number of miRNA targets found annotated in each category over the total number of recognized targets indicated in round brackets).

To understand which could be the common molecular pathways linking the two frailty domains, we performed a functional enrichment analysis of target genes of these 10 selected miRNAs. Significant enrichment was found for miR-21-3p, miR-206 and mir486a-5p and the relevant pathways, as shown in Figure 7.

These results deserve a brief discussion. PTEN (Phosphatase and Tensin Homolog), which is the master gene tagged by eight of the candidate miRNAs, encodes for a multi-functional phosphatase belonging to the PI3K/AKT/mTOR pathway. This evolutionarily conserved component, most prominently known for its function in tumorigenesis, is increasingly seen as having a metabolic role as a negative regulator of the IIS (Insulin Signaling pathway) [165].

In humans, recent results indicate a role of PTEN in the control of adipose tissue growth with aging in cultured cells, associated with a higher insulin sensitivity [166] and insulin resistance in the pathogenesis of T2D [167]. Interestingly, the KEGG analysis showed an enrichment of molecular pathways related to nutrient regulation, such as insulin resistance, FOXO signaling, biosynthesis of amino acids and penthose phosphate pathway. Among the miRNAs prioritized by the KEGG analysis, miR-21, a PTEN inhibitor and a downstream effector of AKT, is reported to be able to reverse high glucose- and high insulin-induced Insulin Resistance in 3T3-L1 adipocytes, through modulating the PTEN-AKT pathway [168].

Moreover, as previously stated, miR-21 is, an inflamm-miRNA particularly linked to sarcopenia [67] but with a relevance in human age-related diseases [116].

The importance of inflammation as a crucial pathway in frailty comes from the interrogation of the STRING database (https://string-db.org/ accessed on 11 July 2022) showing that PTEN is functionally interrelated with two further significant targets of the miRNA-target enrichment: the nuclear phosphoprotein MYC (MYC Proto-Oncogene, BHLH Transcription Factor) and the transcription activator STAT3 (Signal Transducer and Activator Of Transcription 3). This trio is part of an inflammatory regulatory network, particularly linked to the downstream program of IL6, which exerts both pro- and anti-inflammatory activities, eliciting different biological responses on different cell types [169,170,171]. Overall, these evidences further confirm the relevance of the energetic metabolism and inflammation pathway in human frailty. This observation is further confirmed by the other two miRNAs merged from the KEGG analysis, miR-206 and miR-486-5p. Known as myomiRNA, miR-206 suppresses IGF1 [172] and regulates also inflammation [173]. In turn, miR-486-5p inhibits inflammatory response [174] and is known to up-regulate the expression of silent information regulator 1 (SIRT1), a major regulator of lifespan and metabolic disorders [175], whose levels have been associated with frailty in older adults [176]. Thus, it appears very probable that these miRs may represent the link between nutrition and inflammation as master regulators of frailty.

## 6. Conclusions and Final Remarks

The study of miRNAs represents an emerging area of interest in the aging research. For their possibility to target several biological processes, they may represent efficient diagnostic and prognostic biomarkers for frailty, which is characterized by impairment in multiple domains, likely sharing common molecular pathways. Here we investigated this issue by reviewing the literature on miRNAs associated with phenotypes of physical and cognitive dysfunctions, with the aim of identifying regulators common to both frailty domains. A target enrichment and functional enrichment approach provided us with a panel of 10 miRNAs, which target energy metabolism and inflammatory pathways, further confirming the relevance of these mechanisms in the failure of homeostasis related to frailty, and which could be tested as signatures of impairment in both frailty areas.

Although the study of differentially expressed miRNAs in frailty is at its infancy and further studies are necessary, we are very confident that the combination of miRNA-panels and traditional biomarkers may have a clinical value, representing a promising tool for the screening of the population at risk of frailty, to reach the aim of modeling health trajectories toward positive outcomes.

## Figures and Tables

**Table 1:** Gene targets significantly associated with the 21 miRNA markers candidates for the in silico analysis.

miRNA-Target Enrichment Results												
Gene	*p*-Value	FDR	O Rt	Interacting miRs	miR 1	miR 2	mioR 3	miR 4	miR 5	miR 6	miR 7	miR 8
*PTEN*	2.75 × 10^−^^6^	0.011	0.126	8	miR-26a	miR-29b	miR-23a	miR-92a	miR-155	miR-34a	miR-486	miR-21
*ATP1A1*	3.02 × 10^−5^	0.016	0.048	4	miR-93	miR-92a	miR-26a	miR-155				
*CIT*	4.20 × 10^−5^	0.016	0.052	4	miR-93	miR-29b	miR-92a	miR-486				
*GATA3*	1.59 × 10^−5^	0.016	0.018	3	miR-92a	miR-29b	miR-27a					
*GIT2*	3.70 × 10^−5^	0.016	0.024	3	miR-26a	miR-210	miR-92a					
*MYC*	2.84 × 10^−5^	0.016	0.140	7	miR-26a	miR-30a	miR-92a	miR-23a	miR-125a	miR-34a	miR-29b	
*PPP1R2*	4.20 × 10^−5^	0.016	0.052	4	miR-210	miR-142	miR-30a	miR-34a				
*RGS5*	3.13 × 10^−5^	0.016	0.079	5	miR-142	miR-92a	miR-124	miR-9	miR-23a			
*STAT3*	2.64 × 10^−5^	0.016	0.107	6	miR-92a	miR-874	miR-21	miR-23a	miR-124	miR-29b		
*TKT*	3.70 × 10^−5^	0.016	0.024	3	miR-92a	miR-26a	miR-206					
CAMKV	6.57 × 10^−5^	0.020	0.058	4	miR-92a	miR-26a	miR-23a	miR-874				
CCL8	6.22 × 10^−5^	0.020	0.008	2	miR-23a	miR-92a						
PDS5B	6.22 × 10^−5^	0.020	0.008	2	miR-27a	miR-92a						
CTC1	7.08 × 10^−5^	0.020	0.127	6	miR-93	miR-92a	miR-26a	miR-29b	miR-181a	miR-874		
COL4A2	9.55 × 10^−5^	0.024	0.032	3	miR-29b	miR-92a	miR-210					
HES1	9.55 × 10^−5^	0.024	0.032	3	miR-23a	miR-92a	miR-9					
CPEB4	1.07 × 10^−4^	0.025	0.101	5	miR-26a	miR-34a	miR-92a	miR-874	miR-27a			
KIAA1671	1.25 × 10^−4^	0.026	0.068	4	miR-93	miR-29b	miR-92a	miR-30a				
LDHB	1.23 × 10^−4^	0.026	0.0350	3	miR-186	miR-23a	miR-210					
CNOT1	1.94 × 10^−4^	0.029	0.040	3	miR-93	miR-92a	miR-23a					
CSAG1	1.85 × 10^−4^	0.029	0.012	2	miR-186	miR-93						
ESR1	2.04 × 10^−4^	0.029	0.116	5	miR-206	miR-29b	miR-26a	miR-142	miR-874			
FUK	1.85 × 10^−4^	0.029	0.012	2	miR-93	miR-92a						
MCM3	1.56 × 10^−4^	0.029	0.037	3	miR-93	miR-92a	miR-210					
P2RX7	2.15 × 10^−4^	0.029	0.078	4	miR-9	miR-146a	miR-186	miR-125a				
PFDN2	1.56 × 10^−4^	0.029	0.037	3	miR-93	miR-210	miR-92a					
PIK3CG	2.15 × 10^−4^	0.029	0.078	4	miR-29b	miR-27a	miR-142	miR-26a				
PSMC3	1.85 × 10^−4^	0.029	0.01	2	miR-92a	miR-23a						
WDR77	1.94 × 10^−4^	0.029	0.076	4	miR-27a	miR-93	miR-186	miR-125a				
PPARD	2.38 × 10^−4^	0.031	0.043	3	miR-92a	miR-29b	miR-30a					
KCTD5	2.89 × 10^−4^	0.036	0.084	4	miR-92a	miR-26a	miR-125a	miR-34a				
TET2	2.87 × 10^−4^	0.036	0.045	3	miR-92a	miR-29b	miR-26a					
APC	3.43 × 10^−4^	0.037	0.048	3	miR-210	miR-27a	miR-142					
CPEB2	3.47 × 10^−4^	0.037	0.088	4	miR-210	miR-26a	miR-92a	miR-142				
FAU	3.69 × 10^−4^	0.037	0.016	2	miR-92a	miR-23a						
GPD1L	3.69 × 10^−4^	0.037	0.016	2	miR-210	miR-142						
IRAK1	3.43 × 10^−4^	0.037	0.048	3	miR-93	miR-92a	miR-142					
PHB	3.69 × 10^−4^	0.037	0.016	2	miR-27a	miR-26a						
SCAF8	3.69 × 10^−4^	0.037	0.016	2	miR-29b	miR-92a						
VMAC	3.43 × 10^−4^	0.037	0.048	3	miR-146a	miR-186	miR-125a					
HMGCR	4.05 × 10^−4^	0.039	0.051	3	miR-92a	miR-29b	miR-27a					
HECTD1	4.74 × 10^−4^	0.043	0.053	3	miR-210	miR-142	miR-92a					
TBC1D16	4.74 × 10^−4^	0.043	0.053	3	miR-26a	miR-186	miR-210					
TUT1	4.74 × 10^−4^	0.043	0.053	3	miR-93	miR-92a	miR-26a					
ABCB9	6.12 × 10^−4^	0.046	0.020	2	miR-210	miR-26a						
CDH1	6.34 × 10^−4^	0.046	0.059	3	miR-92a	miR-23a	miR-9					
DUSP5	5.51 × 10^−4^	0.046	0.056	3	miR-27a	miR-92a	miR-26a					
IL6	6.34 × 10^−4^	0.046	0.059	3	miR-142	miR-26a	miR-125a					
INPP5A	6.12 × 10^−4^	0.046	0.020	2	miR-210	miR-142						
KIF20A	6.12 × 10^−4^	0.046	0.020	2	miR-92a	miR-23a						
NEK6	6.12 × 10^−4^	0.046	0.020	2	miR-92a	miR-23a						
PDE4B	6.12 × 10^−4^	0.046	0.020	2	miR-26a	miR-34a						
SOCS6	6.34 × 10^−4^	0.046	0.059	3	miR-23a	miR-27a	miR-142					
UBE2R2	6.12 × 10^−4^	0.046	0.020	2	miR-93	miR-92a						
ZNF618	6.34 × 10^−4^	0.046	0.059	3	miR-21	miR-27a	miR-210					

Significance was considered under a level of FDR < 0.05, and a minimum number of 2 interactions gene-miRNAs. In italics are indicated the 10 top-ranked targets (*p* < 4.5 × 10^−5^). FDR: False Discovery Rate; OR: Odd ratio.

## Data Availability

Not applicable.

## Supplementary Materials

The following supporting information can be downloaded at: https://www.mdpi.com/article/10.3390/biology11081151/s1, Table S1: A summary of microRNAs (miRNAs) associated with pathways related to physical frailty in human; Table S2: A summary of microRNAs (miRNAs) associated with pathways related to cognitive frailty in humans.
