# Supplementary material for: Biomarkers of Frailty: miRNAs as Common Signatures of Impairment in Cognitive and Physical Domains
Source: Biology (Basel). 2022 Jul 31;11(8):1151. doi: 10.3390/biology11081151 (PMC9405439; doi:10.3390/biology11081151)
Supplement: Supplementary file 1 [file biology-11-01151-s001.zip › Table S1_proofread_ok.pdf]

Table S1: A summary of microRNAs (miRNAs) associated with pathways related to physical frailty in humans

**MiRNA**	**Function in physical/*cognitive* domains**	**Direction of regulation**	**References**
miR-1	Promoter of skeletal muscle differentiation and regeneration	Decreased in muscle impairment<br>Increased after acute exercise	[72]<br>[73]
**miR-9 (miR-9-1, miR-9-2, and miR-9-3)**	Inhibitor of Skeletal Muscle Satellite Cell Proliferation	Decreased after acute eserecise	[73,74,75]
miR10a-5p<br>miR10a-3p	Inhibitor of myogenic differentiation<br>Negative Regulator ossification	Increased in osteoporosis<br><br>Increased in muscle loss	[76]<br><br>[68]
miR-19a/b	Inflammatory responses, muscle cell apoptosis; fat-free mass metabolism	Increased in sarcopenia	[77,78]
**miR-21**	Muscle atrophy following denervation; muscle regeneration; myogenic differentiation of satellite cells; inflamm-miR; mitomiR	Increased in sarcopenia<br><br>Increased levels led to fewer and smaller myotubes in satellite cells	[67]<br><br>[79]
miR-21-5p	Osteogenic differentiation	Decreased in osteoporosis<br>Increased in osteoporosis	[80]<br>[81,82]
miR-22-3p	Smooth muscle cell differentiation; Regulation of endothelial cell proliferation	Increased in osteoporosis	[76]
**miR-23a-3p**	Inhibitor of myogenic differentiation	Increased in osteoporosis;<br>Increased in muscle impairment;<br>Decreased after acute exercise	[81]<br>[72]<br>[73]
miR-24-3p	Inhibitor of myogenic differentiation	Increased in osteoporosis;	[81]
**miR-26a**	Promoter of myogenic differentiation	Increased in skeletal muscle regeneration after injury	[83]
**miR-27a**	Satellite cell proliferation; Promoter of myoblast differentiation; mitomiR	Overexpression in muscle prevents diabetes-induced muscle cachexia	[84]

**miR-29b**	Myotube formation; myoblast proliferation; mitomiR	Increased in muscle atrophy cellular models	[85]
**miR-30a-3**	Skeletal muscle protein turnover and atrophy	Increased in muscle atrophy cellular models	[86]
miR-31	Regulation of myogenic transcription factors; mitomiR	Decreased after acute exercise	[73]
**miR-34a/c**	Muscle protein synthesis; targeting of cell senescence factors; mitomiR<br>Myoblast proliferation and differentiation	Increased in sarcopenia<br><br>Increased during myogenesis in muscle cellular model	[84]<br>[87]
**miR92a-3p**	Oxidative stress; endothelial dysfunction; mitomiR	Increased in physical decline	[68]
**miR-93**	Inhibitor of myogenic differentiation	Increased in osteoporosis	[81]
miR-100-5p	Inhibitor of myogenic differentiation	Increased in osteoporosis	[81]
miR-122a-5p	Inhibitor of myogenic differentiation	Increased in osteoporosis	[81,82]
**miR-124**	Inhibitor of myogenic differentiation; inflamm-miR	Increased in osteoporosis	[72]
**miR-125b**	Inhibitor of myogenic differentiation	Increased in osteoporosis<br>Decreased in muscle impairment	[72,81,82]
miR-133a/b	Satellite cell proliferation; Myoblast differentiation	Decreased in sarcopenic patients;<br>Increased in osteoporosis<br><br>Decreased in osteroporosis and muscle impairment;<br>Increased after acute exercise<br>High levels protective for muscle atrophy	[80,88,89,90]<br><br>[72,76];<br><br>[73]<br>[91]
miR-139-5p	Myogenic differentiation; cell growth	Decreased in osteogenic differentiation in human mesenchymal cells	[92]
**miR-142-3p**	Osteoblast differentiation	Increased in osteoblast differentiation in human mesenchymal cells	[93]
miR-142-5p	Muscle homeostasis	Down-regulated in frailty subjects	[69]

miR-143-3p	Inhibitor of myogenic differentiation	Upregulated in hypertrophic response in human skeletal muscle	[94]
**miR-146a**	Promoter of satellite cell differentiation; inflamm-miR	Dysregulated in muscle disorders;<br>Increased in osteoarthritic patients;<br>Down-regulated in sarcopenia	[95 (and references therein)]<br>[89]
miR-148a-3p	Inhibitor of myogenic differentiation	Increased in osteoporosis	[81]
miR-151a	Cell proliferation; mitomiR	High levels protective for muscle atrophy	[91]
**miR-155**	Inhibitor of myogenic differentiation; inflamm-miR; mitomiR	Increased in aged muscle cells;<br>Down-regulated in sarcopenia	[96]<br>[89]
**miR-181 (miR-181a- miR-181b- miR-181c)**	Myogenic differentiation; inflamm-miR; mitomiR	Decreased in aged muscle;<br>Overexpression in osteogenesis<br>Increased after acute exercise	[97]<br>[51]<br>[73]
miR185-3p	Cell migration; angiogenesis; regulation of LDL clearance	Increased in physical decline	[68]
**miR-186**	Muscle differentiation, development and muscular atrophy	Increased levels decrease myogenin expression in primary muscle cells;	[98]
miR-187	Promoter of myogenic differentiation	Decreased in osteoporosis	[99]
miR-194-3p<br>miR-194-5p	Muscle atrophy; lipid accumulation in skeletal muscle cells; myogenic differentiation	Increased in muscle loss;<br>Increased in osteoporosis	[68]<br>[100]
miR-195	Control of satellite cell quiescence; mitomiR	Overexpression of miR-195/497 enhances myogenesis	[101]
miR-203a-3p	Skeletal muscle protein turnover and atrophy	Increased in muscle atrophy cellular models<br>Increased levels correlate with muscle mass index and intramuscular adipose tissue content	[86]<br>[102]
**miR-206**	Myoblast differentiation and regeneration	Decreased in muscle impairment;<br>High levels protective for muscle atrophy	[67,72,91]

miR-208a/b	Promoter of myogenic differentiation; specification of muscle fibres	Increased levels in response to endurance exercise in healthy men;<br>Down-regulated in sarcopenia	[103]<br><br>[89]
**miR-210**	Inflamm-miR; overexpression protects from muscular damage	Down-regulated in sarcopenia	[89]
miR-222	Differentiation of skeletal muscle cells	Down-regulated in sarcopenia	[89]
miR-326	Cell proliferation and apoptosis	Increased in muscle loss	[68]
miR-328	Promoter of myogenic differentiation	Decreased in osteoporosis;<br>Down-regulated in sarcopenia	[76]<br>[89]
miR-422a	Regulation of cell growth and proliferation	Increased in osteoporosis	[104]
miR424a-5p	Regulation of cell growth and proliferation	Increased in muscle wasting	[105]
miR-431	Myogenic differentiation and muscle regeneration	Decreased levels in aged myoblast	[106]
miR-449b-5p	Cell cycle control and differentiation	Increased levels in old muscle samples	[51, 78]
**miR-486**	Myoblast differentiation; muscle atrophy	Decreased in muscle impairments	[72,95]
miR-489	Control of satellite cell quiescence	Overexpression suppresses muscle regeneration	[107]
miR-497	Control of satellite cell quiescence	Overexpression enhances myogenesis in cellular models	[101]
miR-499	Biomarker of muscle atrophy; mitomiR	Down-regulated in sarcopenia	[89]
miR-503	Promoter of myogenic differentiation	Induced during muscle differentiation in cellular models	[108]
miR-518f	Inhibitor of myogenic differentiation	Increased in osteoporosis	[99]
miR-532-5p	Osteoblast differentiation	Increased in physical frailty;<br>Decreased in osteogenic cell model	[68]<br>[109]
miR-675	Myogenic differentiation	Increased in low muscle mass	[110]
miR-760	Inhibitor of proliferation and differentiation in myoblasts	Increased in muscle loss	[68]
**miR-874**	Osteogenic differentiation	Decreased in osteoporosis	[111]
