# Supplementary material for: Biomarkers of Frailty: miRNAs as Common Signatures of Impairment in Cognitive and Physical Domains
Source: Biology (Basel). 2022 Jul 31;11(8):1151. doi: 10.3390/biology11081151 (PMC9405439; doi:10.3390/biology11081151)
Supplement: Supplementary file 1 [file biology-11-01151-s001.zip › Table S2_proofread_ok.pdf]

Table S2: A summary of microRNAs (miRNAs) associated with pathways related to cognitive frailty in humans

MiRNA	Function in physical/*cognitive* domains	Direction of regulation	References
**miR-9**<br><br>**(miR-9-1, miR-9-2, and miR-9-3)**	Promoter of neural progenitor proliferation CNS development; microglia activation; regulation of Lamina A transcription	Decreased in neurodegenerative traits	[125]
miR-20a	Cell proliferation and differentiation; apoptosis; APP proteolysis	Decreased in cognitive impairment	[126]
**miR-21**	Angiogenesis, cell differentiation and apoptosis; anti-inflammatory	Increased in human glioma cells, in SH-Y5Y cells and in LPS induced macrophages. Increased in CNS disorders	[127]
**miR-23a-3p**	Neuroprotection from β-amyloid toxicity	Decreased in the frontal cortex of MCI subjects	[128]
**miR-26b**	Synaptic maturation;<br>Dendritic branching	Inhibition neuroprotective against oxidative stress	[129]
**miR-27a-3p**	Regulation of NF-kB activity, cholesterol esterification and LDL particles clearance; mitomiR	Decreased in cognitive impairment	[126]
**miR-29c**	Synaptic plasticity; Aβ clearance; dendritic branching; mitomiR	Increased levels associate with preserved cognitive function	[130]
**miR-30b**	Synaptic plasticity	Decreased in cognitive impairment	[131]

**miR-34a/c**	Synaptic transmission; Aβ clearance; Hyppocampal function; neurotransmitter release; dendritic loss; mitomiR;	Increased in age-related inflammatory-neurodegeneration;<br>Increased in learning and memory impairment	[132]<br>[133]
**miR92a-3p**	Synaptic transmission; mitomiR	Increased in schizophrenia, altered synaptic function in MCI	[68,69]
**miR-93**	Angiogenesis; inflammatory response	Increased in vascular impairment	[134]
miR-103a	APP proteolysis	Decreased in cognitive impairment	[126]
**miR-124**	Regulation of alternative splicing in neurons; microglial polarization; inflamm-miRNA	Downregulation inhibits neuroinflammation in cellular models of neurodegeration	[135]
**miR-125b**	Synaptic transmission; neurotransmitter release; tau phosphorylation	Increased levels related to neurotoxicity;<br>Increased in MCI	[136]<br>[137]
miR-128	Modulator of intracellular calcium content	Increased in MCI	[137]
miR-132<br>miR-132-3p	Regulator of cholinergic signaling; synaptic plasticity; tau aggregation; mitochondrial decline	Increased in dementia and decreased in non-demented centenarians<br>Increased levels in MCI patients<br>Decreased levels protect from dementia	[138]<br>[137,139,140],
miR-134	Synaptic plasticity	Downregulation is neuroprotective in cellular models<br>Increased levels in cognitive impairment<br>Increased in MCI	[141]<br>[142]<br>[137]
miR-135	Modulator of the brain's response to physical exercise	Increased in exosome from MCI subjects	[143]

miR-137	Neuronal cell proliferation and differentiation; Pre-sinaptic plasticity	Overexpression upregulated target genes related to synaptic modulation in cognitive and psychiatric disorders	[144]
miR-138-5p	Synaptic plasticity; tau phosphorylation	Overexpression associates to decreased memory performance	[145]
miR-140-5p	Negative regulator of ADAM10 and its transcription factor SOX2, activated by Aβ	Predictor of cognitive decline<br>Up-regulated in dementia	[146]<br>[147]
**miR-142-3p**	Regulatory T Cell Function; cell proliferation	Increased in different kind of dementia	[131]
**miR-146a**	Regulator of the NF-κb pathway; tau phosphorylation; inflamm-miRNA;	Increased in neurodegenerative conditions;<br>Overexpressed in MCI	[120]<br>[148]
**miR-155**	Neuroinflammation; microglial activation; regulation of immune response; inflamm-miR; mitomiR	Increased in neurodegenerative conditions	[120]
**miR-181**<br>**(miR-181a-miR-181b-miR-181c)**	Synaptic plasticity; neuroinflammation; Aβ clearance; apoptosis; inflamm-miR; mitomiR	Overexpression in MCI patients who later convert to AD	[148]
**miR-186-5p**	Synaptic signalling; Amyloid-beta formation	Upregulated in AD pathogenesis	[149]
miR-193b	Regulator of APP	Increased in exosome from MCI patients	[143,150]
miR200a-3p	Aβ accumulation, apoptosis	Increased in different kind of dementia	[131]
miR-204	Cell migration and differentiation; microglial activation; IL6 production; prostaglandin byosinthesis	Down-regulated in hyppocampal sclerosis<br>Upregulated in serum samples from patients affected by neurodegenerative disorders	[151]<br>[152]

**miR-206**	Synaptic maturation	Increased levels in MCI patients	[139]
**miR-210**	Vesicular trafficking; synaptic plasticity; Inflamm-miR;	Decreased levels correlate with increase in MCI severity	[153]
miR-212	Neuroprotection from Aβ-amyloid toxicity	Decreased levels protect from dementia	[140]
miR-218	Synaptic trafficking	Down-regulated in hyppocampal sclerosis	[151]
miR-223	Granulocyte differentiation and myeloid progenitor proliferation.	Increased in neurodegenerative conditions	[120]
miR-323-3p	Amyloid precursor protein biosynthesis	Increased in MCI	[137]
miR-382	Cell proliferation and angiogenesis	Increased in MCI	[137]
miR-384	Cell proliferation and angiogenesis	Increased in exosome from MCI patients	[143]
miR483-5p	Synaptic plasticity	Increased in different kind of dementia	[131]
**miR-486-5p**	Synaptic plasticity	Increased in different kind of dementia	[131]
miR-491-5p	Regulation of cell migration	Increased in MCI patients	[137]
miR-501-3p	Synaptic transmission	Decreased levels correlate with lower MMSE	[154]
miR-502-3p	Cell proliferation	Increased in different kind of dementia	[131]
**miR-874**	Cell proliferation	Increased in MCI patients	[137]
